# Factors Affecting Implementation of the California Childhood Obesity Research Demonstration (CA-CORD) Project, 2013

**DOI:** 10.5888/pcd13.160238

**Published:** 2016-10-20

**Authors:** Emmeline Chuang, Julian Brunner, Jamie Moody, Leticia Ibarra, Helina Hoyt, Thomas L. McKenzie, Amy Binggeli-Vallarta, Griselda Cervantes, Tracy L. Finlayson, Guadalupe X. Ayala

**Affiliations:** Author Affiliations: Julian Brunner, University of California, Los Angeles, Fielding School of Public Health, Los Angeles, California; Jamie Moody, Helina Hoyt, Thomas L. McKenzie, Griselda Cervantes, San Diego State University Research Foundation, Institute for Behavioral and Community Health, San Diego, California; Leticia Ibarra, *Clínicas de Salud del Pueblo, Inc.,* Brawley, California; Amy Binggeli-Vallarta, Imperial County Public Health Department, El Centro, California; Tracy L. Finlayson, San Diego State University Research Foundation, Institute for Behavioral and Community Health, and San Diego State University, Graduate School of Public Health, San Diego, California; Guadalupe X. Ayala, San Diego State University Research Foundation, Institute for Behavioral and Community Health, and San Diego State University, College of Health and Human Services, San Diego, California.

## Abstract

**Introduction:**

Ecological approaches to health behavior change require effective engagement from and coordination of activities among diverse community stakeholders. We identified facilitators of and barriers to implementation experienced by project leaders and key stakeholders involved in the Imperial County, California, Childhood Obesity Research Demonstration project, a multilevel, multisector intervention to prevent and control childhood obesity.

**Methods:**

A total of 74 semistructured interviews were conducted with project leaders (n = 6) and key stakeholders (n = 68) representing multiple levels of influence in the health care, early care and education, and school sectors. Interviews, informed by the Multilevel Implementation Framework, were conducted in 2013, approximately 12 months after year-one project implementation, and were transcribed, coded, and summarized.

**Results:**

Respondents emphasized the importance of engaging parents and of ensuring support from senior leaders of participating organizations. In schools, obtaining teacher buy-in was described as particularly important, given lower perceived compatibility of the intervention with organizational priorities. From a program planning perspective, key facilitators of implementation in all 3 sectors included taking a participatory approach to the development of program materials, gradually introducing intervention activities, and minimizing staff burden. Barriers to implementation were staff turnover, limited local control over food provided by external vendors or school district policies, and limited availability of supportive resources within the broader community.

**Conclusion:**

Project leaders and stakeholders in all sectors reported similar facilitators of and barriers to implementation, suggesting the possibility for synergy in intervention planning efforts.

## Introduction

Approximately one-third of US children are overweight or obese (1). Rates are particularly high among Hispanic children and those living in rural communities (1). To more effectively prevent and control childhood obesity, policy makers and practitioners have begun to promote social ecological approaches that simultaneously target changes in multiple sectors and at multiple levels of influence (2).

Preliminary evidence suggests that multisector, multilevel approaches can promote health behavior change and prevent child weight gain (3,4). However, the success of such approaches is contingent on their ability to effectively engage and coordinate activities across diverse community stakeholders (5,6). Differences in community stakeholders’ readiness and willingness to implement policy, system, and environmental changes can significantly affect whether targeted improvements to children’s health and well-being are achieved and sustained (7–9).

We conducted semistructured interviews to identify facilitators of and barriers to implementation experienced by project leaders and stakeholders involved in a multisector, multilevel intervention for childhood obesity prevention and control. Participating stakeholders were located in 3 sectors with high potential to affect childhood obesity — health care, early care and education, and schools — and represented multiple levels of influence within their respective organizations (eg, organizational leaders and frontline staff). Findings contribute to knowledge about how to more effectively coordinate and implement social ecological approaches for obesity prevention and control.

## Methods

Data were drawn from the evaluation of the Imperial County, California, Childhood Obesity Research Demonstration project (CA-CORD) (10). Rates of childhood overweight and obesity in Imperial County are among the highest in California (47% in Imperial County vs 38% in the state overall) (11). Most residents are Hispanic/Latino (83%), and almost one-quarter (24%) live in poverty (12). CA-CORD is 1 of 3 studies funded by the Centers for Disease Control and Prevention to test the effectiveness of integrated health care and public health evidence-based approaches to prevent and control childhood obesity (13). CA-CORD used a 2 × 2 factorial study design to assess changes in body mass index in 1,183 children aged 2 to 11 years assigned to 1 of 4 conditions (health care and public health intervention, health care intervention only, public health intervention only, or control). Intervention activities focused on improving 4 health behaviors: fruit and vegetable consumption, water consumption, physical activity, and sleep. Consistent with a social ecological approach (14), CA-CORD activities spanned multiple sectors (eg, health care, early care and education, schools) and levels of influence (individual, family, organization, and community); cross-sector coordination occurred via a CA-CORD community advisory committee that included members of each sector, some of whom were also members of the Childhood Obesity Prevention Alliance led by the local public health department. A brief overview of CA-CORD intervention activities is provided in Table 1; more detailed information is available elsewhere (11,15).

**Table 1:** Summary of Key Intervention Components in Each Sector,^a^ California Childhood Obesity Research Demonstration Project (CA-CORD), 2013

Sector/Personnel	Intervention Component
**Health care** Providers, medical assistants, patient care coordinator, CHWs, and CHW coordinator	3 largest primary care clinics within 1 federally qualified health center Delivery system design (eg, obesity care team, modifications to electronic health records to facilitate assessment and treatment of childhood overweight and obesity) Practice team preparation including staff and provider training (4.5 hours for providers, 4 hours for staff, 136.5 hours for CHWs) CHW-led family wellness and physical activity workshops (11 hours total per family) based on previous, evidence-based interventions (24–26)
**Schools^b^** Administrators, teachers, school nurses, school wellness committee	All public elementary schools (N = 13) School wellness policy change SPARK (19) (3–6 hours of training and curriculum access) BMI measurement (4–8 hours of training) Structural water promotion Sleep curriculum and tip sheets Parent outreach (eg, letter tailored to child BMI) Social marketing campaign
**Early care and education centers^c^** Directors, providers	Early care and education centers in 4 agencies (N = 13) NAP SACC (27) Wellness policy change SPARK (19) (7 hours of training and curriculum access) Quarterly trainings (3 hours each) and technical assistance Physical activity equipment Cooking toolkits Social marketing campaign
**Community** Coalition coordinator, public health officials, parks and recreation coordinators, restaurant managers/owners	CA-CORD Advisory Committee that included members of COPA, which is led by local public health department (quarterly meetings to raise awareness of activities in each sector) Community-level social marketing campaign

Abbreviations: BMI, body mass index; CHW, community health worker; COPA, Imperial County Childhood Obesity Prevention Alliance; NAP SACC, Nutrition and Physical Activity Self-Assessment for Child Care; SPARK, Sports, Play, and Active Recreation for Kids.

a This study focused only on facilitators of and barriers to implementation experienced by key stakeholders in the health care, schools, and early care and education sectors. Data on community recreation departments are not included, because intervention activities were being conceptualized during intervention year one; data on restaurants and on factors affecting family engagement with CA-CORD are described elsewhere (28,29).

b To be eligible for CA-CORD, school-aged children needed to attend one of these schools.

c Early care and education intervention activities were conducted in 2 temporally distinct waves; this study includes only the 13 early care and education centers that participated in intervention year-one CA-CORD activities.

A multiple holistic case study design was used, with participating organizations as the unit of analysis (16). Of the 29 organizations from the health care, early care and education, and school sectors participating in CA-CORD during fiscal year 2013, 27 agreed to participate in this study. We interviewed 68 key stakeholders from these organizations (25 from health care, 17 from early care and education, and 26 from schools), including senior leaders responsible for the decision to participate in CA-CORD (eg, clinic CEO, school superintendents [n = 8]), middle managers and other leaders whose support or participation could affect implementation (eg, principals, clinic managers [n = 30]), and frontline staff directly responsible for implementation (eg, health care and early care and education providers, community health workers, school teachers [n = 30]). CA-CORD project leaders responsible for liaising with organizations to implement CA-CORD activities (n = 6) were also interviewed to provide context about the status of intervention activities in each sector, resulting in a total of 74 respondents.

All interviews were conducted in 2013, approximately 12 months after year-one implementation of CA-CORD intervention activities. Interviews were conducted by trained interviewers in respondents’ language of choice (English or Spanish) using a semistructured interview guide tailored to respondents’ role in the organization. Interview questions (available from the authors upon request) were informed by the Multilevel Implementation Framework (MIF) (15), a conceptual framework of factors affecting implementation of multisector, multilevel approaches. Organization-specific constructs relevant to this study included previous experience promoting healthy behaviors, compatibility with organizational values and priorities, compatibility with existing work processes, leadership support, and implementation climate (ie, the extent to which intervention use is expected and rewarded by the organization). Additional constructs of interest included the characteristics of people involved (ie, frontline staff responsible for implementing CA-CORD and of participating children and/or families), connections to the broader community, and the strength of the external support system (eg, trainings and intervention-specific materials provided by academic-community partners).

On average, interviews lasted from 30 to 60 minutes. With respondents’ permission, most (95%, n = 70) were recorded. For the remaining interviews (n = 4), notes were used in place of a recording.

All recordings were transcribed verbatim. Interviews conducted in Spanish were translated into English by a certified translator. Final transcripts and interview notes were imported into the qualitative software NVivo 10.0 (QSR International) for analysis. We used template analysis (17), in which an initial codebook informed by the MIF was refined to incorporate emergent themes. Initial codes were applied to a subset of 6 transcripts. Coding was compared for consistency by a second person, and the codebook was revised to clarify construct definitions or better highlight critical themes. All transcripts were subsequently coded by 2 investigators. Discrepancies in coding were discussed until consensus was reached. Within-case and between-case analyses focused on the degree to which specific constructs emerged in the data and the degree to which each construct was perceived as affecting implementation. Coded data were also analyzed to identify similarities and differences by sector.

## Results

Three community health care clinics, 13 early care and education centers, and 11 schools agreed to participate in the study. On average, respondents from these organizations were aged 45 years and had been with their organization for 8 years. Most were female (86%) and Latino/Hispanic (69%). All respondents reported facilitators of and barriers to implementing CA-CORD. We summarized major themes according to key MIF constructs (Table 2) and provided illustrative quotations (Table 3).

**Table 2:** Factors Affecting Implementation of California Childhood Obesity Research Demonstration Project (CA-CORD), by Sector, 2013

Factor	Sector		
	Health Care Clinics (N = 3)	Early Care and Education Centers (N = 13)	Schools (N = 11)
**Prior experience promoting healthy behaviors**	Clinics previously distributed educational materials to families but otherwise no experience promoting healthy behaviors among children	10 of 13 centers had previous experience with programs promoting healthy behaviors Prior experience made staff more receptive to CA-CORD Curriculum from other programs may “compete” with CA-CORD activities	All schools had prior experience with programs promoting healthy behaviors Other programs can “compete” with CA-CORD activities Previous implementation failures can generate resistance
**Compatibility with organizational values and priorities**	In all 3 clinics, CA-CORD described as high priority because of high prevalence of chronic disease in the patient population and the importance of preventive care	Six of 13 centers identified CA-CORD as a high priority; only 2 centers described it as a low priority Behavior changes promoted via CA-CORD perceived as beneficial for center staff as well as children	In 5 of 11 schools, CA-CORD described as low priority; only 2 schools identified it as a high priority Perceptions of compatibility strongly affected by respondents’ individual values Perceived compatibility higher for multipurpose activities that address not only physical activity but also positive social interactions
**Compatibility with existing work processes**	CA-CORD activities relatively easy for providers and staff in all 3 clinics to incorporate into daily schedule	Once trained, no difficulty incorporating CA-CORD activities into staff’s daily routine Staff release time to participate in voluntary physical education training can be challenging	CA-CORD activities can be difficult to incorporate into daily schedules given limited time and teachers’ need to focus on academic outcomes
**Leadership support**	High level of leadership support in all 3 clinics Support from senior leadership primarily expressed by permitting providers and staff to participate in CA-CORD Providers described by staff as highly supportive	Leadership supportive of CA-CORD at all 13 centers Support primarily expressed by permitting center staff to participate in CA-CORD	Leadership support highly variable across districts and schools In 3 of 11 schools, new principals were not aware of previous or current programs promoting healthy behaviors
**Implementation climate**	Implementation by providers not recognized or rewarded by leadership Limited data made available regarding clinic performance in assessing or treating overweight or obese pediatric patients	Implementing CA-CORD not required or rewarded by leadership	Implementing CA-CORD not required or rewarded by leadership Consistent, supportive contact from CA-CORD staff can create positive implementation climate even in the absence of more proactive leadership support within the organization
**Characteristics of individuals involved**	Front office staff can assist with distributing promotional materials to families Family engagement significantly affects implementation For low-income families, cost of care, limited time, and lack of transportation are major barriers to engaging in CA-CORD Important to present information in families’ primary language	Child engagement can affect staff’s ability to implement CA-CORD as intended CA-CORD activities could better engage parents to ensure healthy behaviors are reinforced in the home	Teacher buy-in significantly affects CA-CORD implementation and is strongly affected by perceived program benefits and ease of use Many teachers not comfortable implementing physical education and require additional support Parents’ lack of interest can be a barrier to promoting healthy lifestyles in the broader community
**Connection to broader community**	Informational materials should be distributed in many places, not just the pediatric department Some awareness of CA-CORD activities in the broader community	Staff were not aware of broader efforts in the community but thought such efforts were critical for ensuring actual behavior change	Principals in 4 of 11 schools were aware of broader efforts in the community Supportive resources for children who were overweight or obese not always readily available in the community
**External support system**	CA-CORD promotional materials helped reinforce verbal messages from providers and staff Participatory approach in the planning stages of the project important for buy-in	Gradual introduction of CA-CORD activities can prevent staff from being overwhelmed Careful adaptation of CA-CORD activities to match existing resources at each center can help minimize burden on staff Hands-on demonstrations of how to implement CA-CORD activities critical for effective implementation by staff, particularly for SPARK-PE	Regular attendance at staff meetings and/or other follow-up important for obtaining buy-in from teachers Developing a curriculum guide with structured lesson plans can enhance teacher buy-in by making it easier to implement the intervention Resource support particularly important for building teacher comfort with CA-CORD activities related to physical education
**Other facilitators and barriers**	Turnover of community health workers can increase training costs	Turnover in administrators and frontline staff can negatively affect buy-in to the program and the consistency with which it is implemented Smaller centers able to implement CA-CORD more quickly Space constraints can limit ability to implement CA-CORD activities Centers serving prepackaged meals purchased from external vendors cannot control foods served to children	Administrative turnover, particularly of principals, can negatively affect support for CA-CORD activities To accommodate academic scheduling needs, planning for intervention activities must be completed before end of previous academic year

Abbreviation: SPARK-PE, Sports, Play, and Active Recreation for Kids physical education program.

**Table 3:** Illustrative Quotes, by Theoretical Construct, California Childhood Obesity Research Demonstration Project (CA-CORD), 2013

Construct	Quote
**Prior experience promoting healthy behaviors**	1. “We always had movement songs and exercises in previous years, but with SPARK now we follow specific instructions to the songs on the CD.” 2. “Yes, [but] we had to cut other activities in order to implement [CA-CORD]. . . .”
**Compatibility with organizational values and priorities**	3. “This is a high priority for us. We have a high incidence of our population from children to adults that are obese. . . . It’s really a chronic disease issue. . . . Obesity turns into hypertension and diabetes and other issues. . . . It is really a big factor within our organization.” 4. “We are doing it for the children’s benefit . . . for better, healthy, nutritious lives.” 5. “Basically, what teachers are held accountable for, what they feel most strongly about in terms of teaching and their expected outcomes, is math, reading, language arts . . . that’s where the priority is.”
**Compatibility with existing work processes**	6. “For me, [CA-CORD] is less than I was doing prior. Before, I was trying to do everything on my own. Now I can say, ‘Here, I have help!’ . . . and I’m not doing everything on my own. It saves me time, maybe an hour or two per week.” 7. “I still see patients as usual . . . nothing [changes] except putting in the referrals . . . an hour a week, I guess.” 8. “PE is outside a lot of teachers’ comfort zones.”
**Leadership support**	9. “Our principal wants us to try [CA-CORD]. . . . She’s definitely very supportive. . . . She’s always asking ‘Do you need anything? How’s it going? Do you need more training?’ You just know she’s there if needed.”
**Implementation climate**	10. “No one comes to me once a week and says, ‘This is what we need to do, this is what we need to improve.’ No one has come to me with this information.” 11. “Some teachers didn’t even take their kids out to PE. Even though it was education code, they would skip it completely. There’s no follow-through from administration to make sure teachers do what they’re supposed to do.” 12. “[CA-CORD staff] kept checking up with us every month or so to see how we were doing in and present to the staff, so, yes, it felt like we were expected to participate.”
**Characteristics of individuals involved**	13. “Some teachers really gung-ho. They’re enjoying it, they like it. And others are like, ‘Oh no, another program, another thing to do.’ . . . We’ve got one, she’s all gung-ho on it, and she’s got us all going.” 14. “We did well with superintendents and principals, but where we missed the boat initially was coordinating with teachers and nursing staff. . . . They never got the communication from district administration, and they were the ones that were going to be crucial for actually implementing project activities.” 15. “You have to have your parents on board. A major factor for this project, the main thing that will either be successful or unsuccessful, is the parent participation with the children.” 16. “I think it’s good that parents be included in children’s activities, so they know what the program is about. . . . I don’t know if you could include these activities in a parent conference or staff–parent meeting, include activities they can do with the children at home.” 17. “Families are low-income . . . it’s harder for them, plus the schedules, a lot of families work out in the fields . . . they’re not going to be wanting to come . . . it’s hard for them.” 18. “Some of those children . . . they don’t always participate in all the activities we offer, and we can’t force them. . . . We offer it, we encourage them, but if they don’t do there’s nothing we can do . . . ”
**Connection to broader community**	19.“Right now, the public health department only has one nutritionist, so it’s not enough for the community . . . ” 20. “The [families] that wanted to get resources, we didn’t have enough to send them to . . . we didn’t really solve the true problem in getting them help. . . . We don’t have buy-in from the private pediatricians, and we don’t have resources locally . . . ” 21. “The school nurse mentioned she would send out the referral, and then . . . the pediatrician would tell them ‘Oh, you don’t really have a problem’ . . . and the parents were upset with the nurse . . . so we didn’t really have that collaborative support . . . ”
**External support system**	22. “Teachers are not PE specialists. They were trained to teach the academics, so it’s nice to bring people in that are PE credentialed to provide that staff development, teach lessons, provide lesson plans for teachers to be able to do with the kids.” 23. “In the beginning it was hard. As we became more familiar with [CA-CORD], our contact would say, ‘If you guys have any difficulty . . . if you don’t understand it, let me know and I’ll come and teach you.’ That was helpful.”

Abbreviations: CD, compact disc; PE, physical education; SPARK, Sports, Play, and Active Recreation for Kids.

### Previous experience promoting healthy behaviors

Previous experience promoting healthy behaviors varied across sectors. In the clinic setting, providers and staff reported distributing educational materials to families but otherwise did not have prior experience promoting healthy behaviors among pediatric patients. For these providers and staff, CA-CORD was viewed as providing important, additional resources that supported their work with children and families. By contrast, respondents in most early care and education centers (10 of 13) and all schools (11 of 11) had prior experience implementing programs to promote healthy behavior, such as Head Start’s I Am Moving, I Am Learning initiative (18). For staff in these centers and schools, CA-CORD was often perceived as supplementing existing curriculum by providing additional, structured activities they could engage in with the children (Table 3, quotation 1). In a few cases, these activities were perceived as competing with other programs (quotation 2). In several schools, a previous failed effort by the district to implement the SPARK (Sports, Play, and Active Recreation for Kids) physical education program (SPARK-PE) (19), because of insufficient teacher training, was identified as contributing to teacher resistance to implementing CA-CORD.

### Compatibility with organizational values/priorities and existing work routines

In general, respondents in the health care and early care and education sectors described CA-CORD as highly compatible with organizational priorities (Table 3, quotations 3 and 4). Perceptions of CA-CORD were more mixed in schools; respondents in 5 of 11 schools identified CA-CORD as a low priority for their organizations. Primary reasons given for this low rating included competing demands and a need for teachers to focus on academic outcomes for which they were held accountable, such as reading and math (quotation 5). Respondents who rated CA-CORD more highly typically perceived a greater association between healthy behaviors and successful learning or felt that activities achieved multiple purposes (eg, improved both physical health and cooperative social behavior).

Perceptions of CA-CORD’s compatibility with existing work routines also varied across sectors. In the health care sector, respondents described CA-CORD as highly compatible with their existing work routines and not particularly time-consuming to implement (Table 3, quotations 6 and 7). In schools and to a lesser extent in the early care and education sector, CA-CORD activities were described as time-consuming to learn and difficult to implement given competing demands on teachers’ and providers’ time. This perception was particularly true for SPARK-PE activities, which were often outside teachers’ and providers’ comfort zones and viewed as more difficult to implement than other CA-CORD activities (quotation 8).

### Leadership support and implementation climate

Senior leaders in all 3 sectors were generally supportive of CA-CORD. However, this support was typically passive, with the most commonly reported indicator being permission to participate in CA-CORD activities. The major exception was the superintendent of one school district who was heavily involved in district wellness committee meetings and willing to allocate significant resource support for CA-CORD. Many respondents within this district identified this resource support, which included funds to hire a part-time physical education support staff member, as a valuable facilitator to project implementation.

Respondents indicated that middle managers, such as early care and education directors and school principals, varied in their support for the project. For example, in several schools, principals were described as actively engaged in promoting CA-CORD, for example, by frequently interacting with teachers to ensure they had the support needed to implement intervention activities (Table 3, quotation 9). In other schools, principals either took no action or engaged in behaviors that negatively affected implementation (eg, in one case by reprimanding a teacher who allowed students to leave the classroom to get a drink of water).

In all 3 sectors, respondents indicated that engaging in CA-CORD activities was not expected or rewarded by leaders in their organizations (Table 3, quotation 10). This perception was particularly strong in the school sector. For example, even though California’s education code requires that students engage in 200 minutes of physical education every 10 school days, several respondents admitted that teachers often did not achieve this requirement and that conformity to education code requirements was not enforced by leadership (quotation 11). However, multiple respondents also indicated that regular, supportive contact from CA-CORD staff during training sessions and staff meetings created a positive implementation climate even in the absence of more proactive leadership support and follow-up within the organization (quotation 12).

### Characteristics of frontline staff and children and families

Respondents in the health care and early care and education sectors identified the supportive attitudes of frontline staff as facilitating the implementation of CA-CORD activities. In schools, teacher buy-in was inconsistent and served as either a barrier or a facilitator, depending on whether teachers resisted or championed the project (Table 3, quotation 13). Consequently, CA-CORD staff reported needing to allocate time to engage teachers as well as principals (quotation 14).

Respondents in all 3 sectors identified parent engagement (or lack thereof) as significantly affecting implementation, because it affected whether healthy behaviors were reinforced in the home (quotations 15 and 16). Although some respondents reported ongoing efforts to engage parents (eg, by distributing materials that would allow parents to try CA-CORD activities at home), most simply identified lack of parental engagement as a barrier to improving targeted health behaviors. In the health care setting, several respondents identified income and language barriers as contributing to lack of parent engagement in CA-CORD (quotation 17).

In 2 of the 3 sectors (early care and education and school), respondents also identified child engagement as affecting implementation. For example, several teachers and providers noted that they could not force resistant children to participate in SPARK exercises or to try healthy foods (quotation 18). However, in some centers and schools, highly engaged children enhanced teachers’ and providers’ enthusiasm for the project and also spurred behavior change in teachers.

### Connection to broader community and external support system

Although CA-CORD project staff identified many initiatives intended to promote healthy behaviors that were taking place in the broader community, most frontline staff were either not aware of them or felt they were still not sufficient. Several respondents expressed frustration that their efforts to promote healthy behaviors were not reinforced by others in the community, either because of limited resources or general lack of support (Table 3, quotations 19–21). Nonetheless, respondents in all sectors felt that connections to the broader community were critical for reinforcing the healthy behaviors promoted by CA-CORD and ensuring sustainable change.

In all 3 sectors, respondents identified technical assistance and support provided by CA-CORD project staff as critical for maintaining project momentum and ensuring activities did not fall by the wayside (quotation 22). Additional facilitators to implementation included the use of a participatory approach by CA-CORD project staff and the decision to gradually introduce intervention activities in a way that would minimally disrupt existing work schedules (quotation 23).

### Other facilitators and barriers

In all sectors, staff turnover was described as a barrier to implementation. In the health care sector, turnover of community health workers contributed to project costs and delayed implementation of educational workshops for families. In the school sector, turnover of principals and other administrative personnel negatively affected leadership support for CA-CORD and necessitated additional effort by CA-CORD staff to re-engage staff at the affected schools. In the early care and education sector, participating centers were all part of large agencies that purposely rotated staff annually. This movement of early care and education providers, supervisors, and even directors was a barrier that had to be taken into account when planning and implementing CA-CORD activities.

## Discussion

Theoretical constructs identified in the MIF were useful for summarizing the major facilitators and barriers experienced by key stakeholders in implementing CA-CORD. Perceptions of the strengths of the external support system and of the importance of parent engagement were remarkably congruent across sectors and consistent with previous research indicating the importance of robust academic–community partnerships and family engagement for health behavior change in rural communities (20). Respondents also consistently emphasized the importance of the broader community for reinforcing health behaviors. Similar to previous literature on innovation implementation (21), study findings confirmed that prior experience with programs promoting healthy behaviors helped strengthen perceived compatibility of CA-CORD with existing work processes. However, particularly in cases of prior failed implementation (eg, in the school district that had previously implemented SPARK-PE with limited success), previous exposure could also increase staff resistance to implementation. Respondents in all 3 sectors identified turnover at multiple levels of the organization as a barrier that should be addressed in the program planning process (eg, by incorporating a train-the-trainer model or other strategies for minimizing knowledge loss due to turnover) (22).

Several sector-specific issues were also identified. In the school sector, lower perceived compatibility of obesity prevention and control activities with organizational priorities contributed to variable leadership support and greater emphasis on the importance of obtaining teachers’ buy-in and support for CA-CORD. Strict scheduling in the school sector also meant that planning for CA-CORD activities needed to be completed by the end of the previous academic year. In the early care and education sector, where centers were often smaller or reliant upon relationships with external vendors to provide services, space constraints and limited control over foods served to children limited staff ability to implement CA-CORD as intended.

In general, however, similarity in the facilitators of and barriers to implementation identified by project leaders and key stakeholders suggest the possibility of common ground in collaborative efforts to develop and sustain social ecological approaches to prevent and control obesity. In particular, findings reinforce the importance of taking a participatory approach during the planning process and of ensuring that proposed changes are introduced in a time frame and manner compatible with stakeholders’ work processes and priorities. Specific actions taken by the CA-CORD team to facilitate implementation included conducting formative assessments to assess organizations’ receptivity to proposed project activities and engaging community members to better understand organizations’ different needs and priorities. Study findings also indicate that the support of senior leaders is necessary but not sufficient for program success; strategies for cultivating buy-in of staff at multiple levels within participating organizations should be considered.

This study had several limitations. First, implementation is often a dynamic, nonlinear process (23). These data provide an overview of key facilitators and barriers to implementation encountered by organizations during the first intervention year of CA-CORD but may not represent a comprehensive list of relevant issues over time. Second, this study focused on a limited number of organizations within a single, rural county in California, which may limit generalizability to other settings. Finally, resource constraints and our desire to minimize respondent burden meant we only interviewed a limited number of people within each participating organization. Although we interviewed a diverse sample of respondents at different levels within each organization and theoretical saturation was achieved (ie, later interviews did not generate new insights to research questions), study findings may not capture all facilitators and barriers encountered during implementation.

Despite these limitations, this study contributes to the literature by capturing the perceptions of project leaders and key stakeholders regarding facilitators and barriers experienced in implementing multilevel approaches to childhood obesity prevention and control. Congruity in perceptions of certain facilitators and barriers represents not only critical points to consider during intervention planning, but also key areas in which stakeholders could fruitfully collaborate in developing and implementing social ecological approaches to obesity prevention and control.
